# Production of thermotolerant, detergent stable alkaline protease using the gut waste of *Sardinella longiceps* as a substrate: Optimization and characterization

**DOI:** 10.1038/s41598-018-30155-9

**Published:** 2018-08-20

**Authors:** Aishwarya Ramkumar, Nallusamy Sivakumar, Ashish M. Gujarathi, Reginald Victor

**Affiliations:** 1 Department of Biology, College of Science, Sultan Qaboos University, PO Box 36, PC 123 Muscat, Oman; 2 Department of Petroleum and Chemical Engineering, College of Engineering, Sultan Qaboos University, Muscat, Sultanate of Oman

## Abstract

The gut wastes of *Sardinella longiceps* were used as substrate for protease production. The gut waste has 61.6% proteins, 21.8% lipids, 8.5% carbohydrates on dry weight basis and trace elements. The significant factors of protease fermentation were screened by Plackett-Burman design. A protease activity of 68.56 U/ml was predicted at 46.31 °C, incubation time 71.11 h, inoculum 4.86% (v/v) and substrate concentration 2.66% (w/v), using response surface methodology. However, the validation experiment showed 73.52 U/ml activity. The artificial neural network was found as a better tool to predict the experimental results. The partially purified protease showed higher activity at pH 9 and 10 and retained 90% activity after 120 h at pH 9. It showed maximum activity at 50 °C and retained 88% residual activity until 90 min at 50 °C. Zn^++^ enhanced the protease activity by 40%. The protease retained an activity of 93, 103, 90 and 98% against urea, β-mercaptoethanol, SDS and tween 80 respectively. The alkaline protease was compatible with all the commercial detergents tested with the residual activity above 90%. The alkaline protease exhibited 22% higher activity on the tryptone soya substrate. The gut waste of *S*. *longiceps* is a worthy low cost substrate for the production of industrially important alkaline protease.

## Introduction

Proteases are extracellular enzymes capable of hydrolysing large protein molecules to smaller peptides or amino acids that can be utilized by microorganisms. Microbes are ideal sources to produce protease enzymes because of their broad biochemical diversity, less complexity in the production process, easy maintenance of organisms, rapid growth, requirement of less space for cultivation and flexibility for manipulation at the genetic level^1^. Bacterial proteases are produced in large quantity due to their specificity, stability and activity in a broad range of physical conditions. Over 60% of the global production of industrial enzymes is proteolytic enzymes and among the proteolytic enzymes, 35% consists of alkaline proteases. These proteases are widely used in different industries such as brewing, baking, cheese-making, cosmetics, detergent, food, pharmaceutical, meat tenderization and leather^2^. In recent years, much interest has been shown in utilizing waste materials as a substrate for various fermentation processes^3,4^. Large amounts of solid and liquid wastes are generated during fish processing and its complex nature makes its disposal complicated and more expensive. Being a rich source of protein, these fish wastes could be utilized as a low cost substrate for the production of microbial proteases^5^.

The protease production is greatly affected by the nutritional and environmental conditions. Hence, it is possible to increment the protease production by manipulating the culture conditions. The required nutrient resources govern the cost of any microbial enzyme synthesis processes. Therefore, finding out a low cost medium and optimization strategies would economically benefit the production process. To accomplish this objective, the Plackett-Burman design (PB) and response surface methodology (RSM) were used in this study to evaluate the positive factors and select optimum conditions of variables for a maximum response. RSM can be used to design experiments, search optimum factors of the responses and evaluate the relative significance of selected factors even in the occurrence of complex interactions among the factors^6^. Further, the artificial neural network (ANN) model is built to test and predict the observed experimental results.

The microorganism, the nature of the substrate and the biochemical features of the enzyme produced are necessary factors to evaluate its biotechnological potential and its possible application in various industrial processes. The biochemical characterization of an enzyme indicates its performance and can forecast its utilization in specific applications^7^. In this study, protease has been produced by a native *Bacillus licheniformis* NK isolated from Nakhl hot spring, Oman using the gut waste of *S*. *longiceps* as an inexpensive substrate. The produced enzyme has also been characterized for its possible industrial applications.

## Results and Discussion

### Physical and chemical factors of sampling site

Totally 101 colonies were isolated from four different sites in Nakhl hot spring, Oman. Physical and chemical parameters recorded at the time of sampling in the four sites, N1, N2, N3 and N4, are given in Table 1. The temperature at the source of the hot spring was 38.8 °C. The pH and dissolved oxygen level of the water increased from the source towards downstream. Calcium and its salts are present in ionic form in hot water and they precipitate out when the temperature decreases. This could be a possible reason for the increase in pH and the dissolved oxygen is never limited in flowing water due to absorption of oxygen from air facilitated by the flow, photosynthetic production of benthic algae and decrease in temperature. The conductivity decreases with the reduction in water temperature because of the reduction in the ionic composition of water. The majority of the microbes was isolated from the soil collected from the sites N3 and N4, which were approximately 50 and 80 meters away from the source. Microorganisms surviving in hot springs have the adaptability to survive in harsh environmental conditions. This ability may be due to their molecular modifications at cellular and sub-cellular levels^8^. Both *Bacillus* spp. and *Brevibacillus* spp. isolated from various hot springs across the world were reported to produce thermostable proteases^9,10^.

**Table 1:** Physical and chemical factors recorded at the sampling sites.

Parameters	N1	N2	N3	N4
Air temperature (°C)	37.5	35.2	37	37
Water temperature (°C)	38.8	37.9	37.7	38.2
pH	7.6	8.3	8.6	8.8
Conductivity (µS)	732	724	705	703
Dissolved oxygen (ppm)	3.05	6.36	7.56	10.3

N1-Source; N2, N3 and N4-downstream sites.

### Composition of gut waste substrate

The composition of gut waste of *S*. *longiceps* is given in Table 2. The gut waste was rich in proteins (61.6%) followed by lipids (21.8%) and carbohydrates (8.5%) on dry weight basis. The essential elements were in the magnitude order of K^+^ > Na^+^ > Ca^2+^ > Mg^2+^ > Fe^2+^ > Zn^2+^ > PO_4_^3−^. Among salts, PO_4_^3-^ dominated the composition followed by Cl^2−^, SO_4_^2−^ and NO_3_^−^. Hence, the gut waste that has rich nutrients and essential elements required for bacterial growth has been considered as a substrate for protease production. It has been previously reported that fish waste comprises of 58% protein, 19% fat and trace amounts of minerals, mainly copper, phosphorus, magnesium, sodium, potassium, calcium, iron, zinc and manganese^11^. Bioconversion of these wastes will help in decreasing the production cost of useful microbial enzymes^12,13^.

**Table 2:** Composition of the gut waste substrate.

Components	mg/g (DW)
Total proteins	616.0 ± 8.32
Total lipids	218.4 ± 5.61
Total carbohydrates	85.11 ± 1.44
Magnesium	0.160 ± 0.01
Calcium	0.230 ± 0.03
Potassium	4.515 ± 0.57
Sodium	1.842 ± 0.08
Phosphorus	0.013 ± 0.00
Iron	0.027 ± 0.00
Zinc	0.020 ± 0.00
Phosphate	5.141 ± 0.83
Chloride	2.751 ± 0.09
Sulphate	0.404 ± 0.06
Nitrate	0.015 ± 0.00

Each concentration is expressed as Mean ± SD; n = 3, DW-dry weight.

### Screening for protease producers

**Figure 1:**
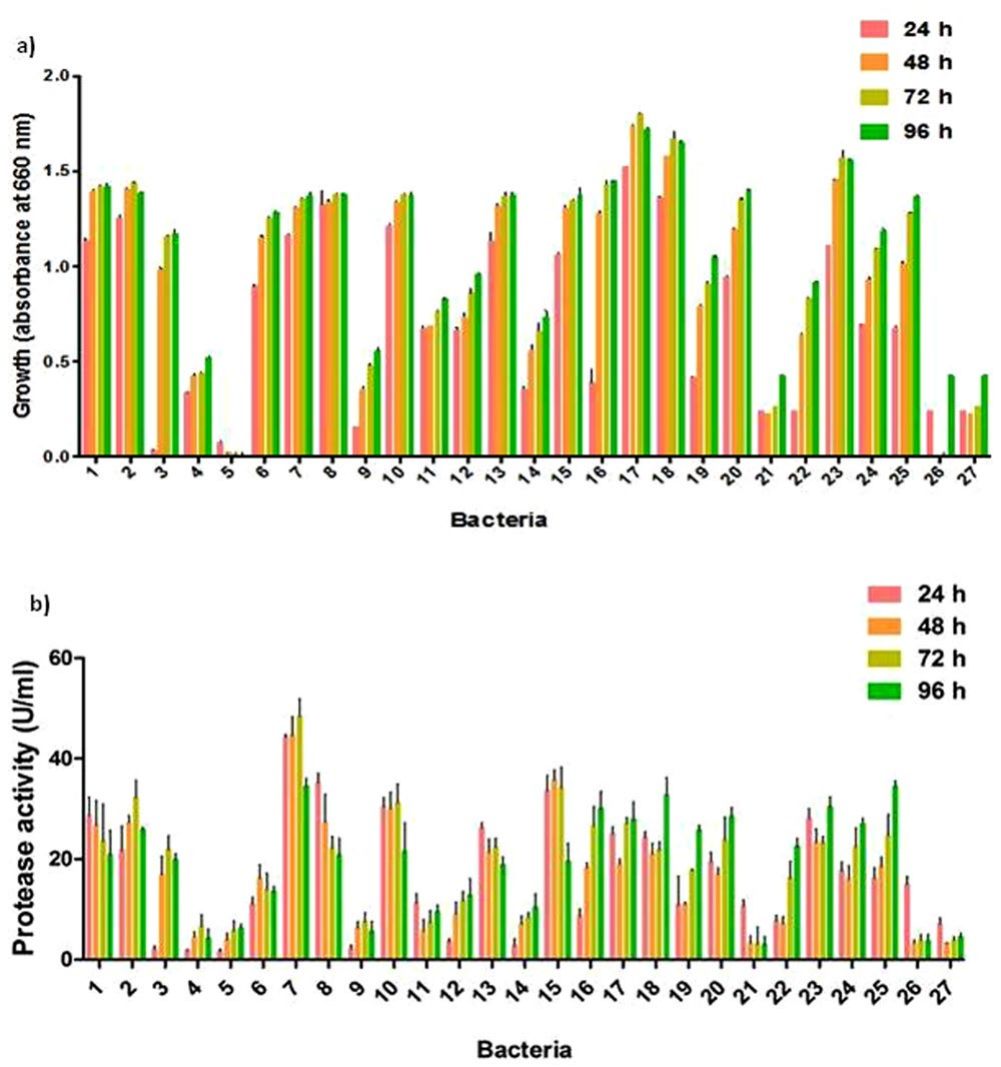
(**a**) Growth and (**b**) protease activity of bacteria in secondary screening. Error bars indicate the standard deviation of the triplicate values.

In primary screening, out of 101 bacterial isolates, 53 colonies produced clearance zones in casein agar. Fish gut waste was used as the substrate for secondary screening. Twenty seven isolates out of 53 were found as best protease producers. In secondary screening, maximum growth was shown by bacterium 17 at 72 h and the least growth was shown by bacterium 5 (Fig. 1a). For the majority of the isolates, growth was maximum at 72 h, after which it enters into stationary phase.

Highest mean protease activity (48.42 units/ml) was exhibited by bacterium 7 at 72 h of incubation (Fig. 1b). One way ANOVA followed by Tukey’s Post Hoc analysis showed that the protease activity of bacterium 7 was significantly higher than all the other bacterial isolates (P < 0.05). This is in agreement with a study that reported on maximum protease production from *B*. *licheniformis* after 72 h in potassium nitrate containing basal medium^14^. It has also been previously reported that alkaline protease from *Bacillus subtilis* Y-108 has exhibited maximum activity at 72 h of incubation in medium containing shrimp and crab shell powder^15^. The extracellular protease production during stationary phase is a characteristic feature of many bacterial species. Three independent experiments were conducted with bacterium 7 using gut waste substrate and it produced maximum activity at 72 h of incubation, consistently. Thus, this bacterium was chosen for optimization and production of protease enzyme. Regression analysis was carried out for growth and protease activity of all the 27 bacteria. A positive relationship between growth and protease activity was observed (R^2^ = 0.73, R^2^ = 0.67, R^2^ = 0.63 R^2^ = 0.74 for 24, 48, 72 and 96 h respectively). This indicates that the bacteria utilized fish gut waste substrate as the primary nutrient for its growth by producing protease enzyme. Positive relationship between growth of bacteria and protease activity has been previously reported^16^.

### Identification of bacteria

**Figure 2:**
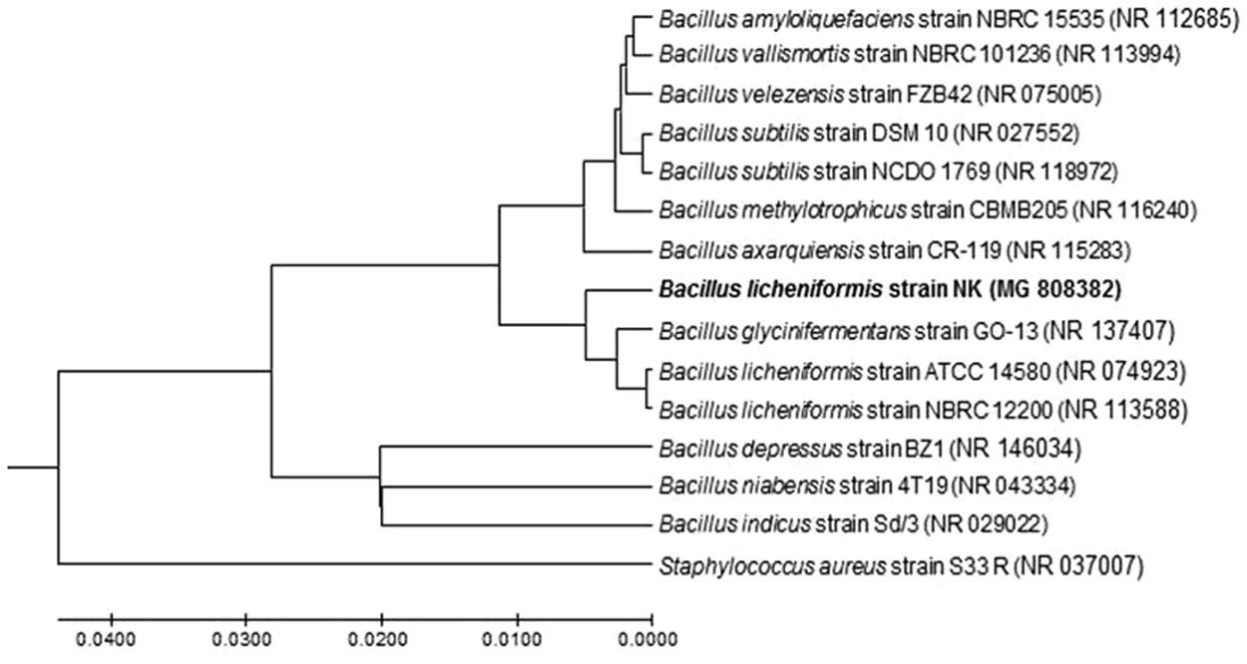
Phylogenetic dendrogram of the relationship between the 16 S rRNA gene sequences retrieved from GenBank and the 16 S rRNA of the *B*. *licheniformis* NK.

All the protease producing bacteria isolated in the secondary screening were identified using MALDI biotyper. The score values greater than 2 and 1.7 indicates a reliable identification at the species and genus levels respectively, whereas the score value less than 1.7 indicates unreliable identification. Among the identified, only one bacterium was identified as *Brevibacillus* spp. and all the other isolates were *Bacillus* spp. The best producer, bacterium 7 was identified as *B*. *licheniformis* with a score of 2.115. Molecular identification of the bacterium 7 was also carried out by 16 S rRNA sequencing and the bacterium was identified as *B*. *licheniformis* NK with the identity similarity of 99% and the error value of zero, thus, confirming the biotyper result. The sequence was submitted to NCBI (accession number: MG 808382). The evolutionary history was inferred using the UPGMA method. The optimal tree with the sum of branch length = 0.18940404 is shown in Fig. 2. The tree is drawn to scale, with branch lengths in the same units as those of the evolutionary distances used to infer the phylogenetic tree. The evolutionary distances were computed using the maximum composite likelihood method and are in the units of the number of base substitutions per site. The analysis involved 15 nucleotide sequences and all the ambiguous positions were removed for each sequence pair. Totally 1574 positions were in the final data set and the evolutionary analyses were carried out using MEGA7.

### Screening of significant factors by PB design

**Figure 3:**
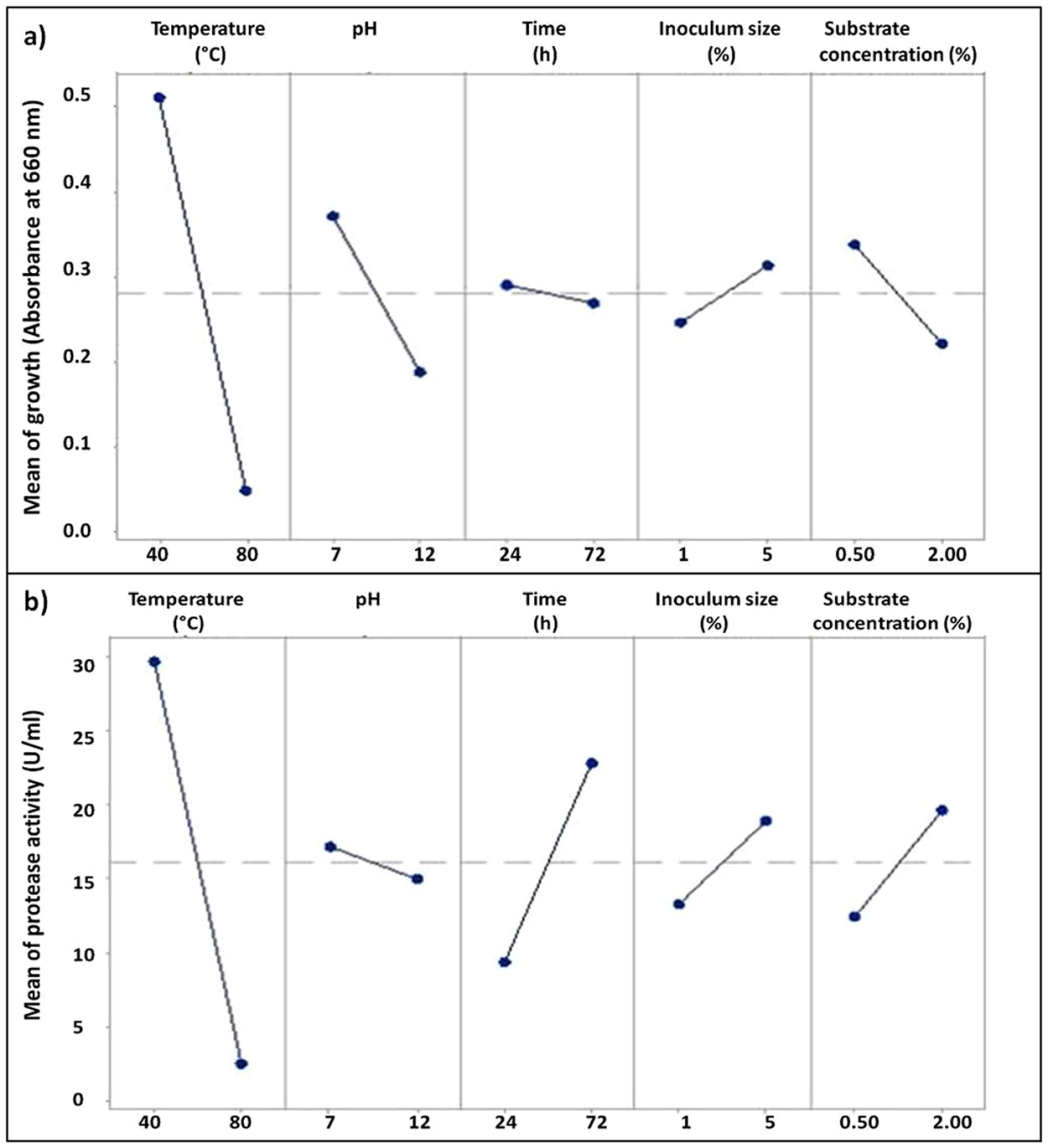
Effect plot showing the effect of factors on (**a**) mean growth and (**b**) mean protease activity of *B*. *licheniformis* in Plackett-Burman design. The points above hatch line have a positive effect and below have negative effects.

The significant variables necessary for the enhanced protease production were screened using the PB design. Five variables, including temperature, pH, time, inoculum and substrate concentration were analyzed (Table 3) for their effects on growth and protease production. The runs generated in PB are shown in Table 4 along with the response variable. The effect of variables on protease activity could be explained with the help of effect plot developed using Minitab version 17. Points above the hatch line have a positive effect and below have a negative effect. The effect of factors in PB design showed that the growth was maximum at 40 °C (Fig. 3a), but the increase in temperature inhibited the bacterial growth. Most favourable pH for growth was 7, and increasing pH decreased the growth. Growth was the maximum at 24 h of incubation and slightly deceased at 72 h. Growth of *B*. *licheniformis* NK was increased with the increasing concentration of inoculum and was maximum at 5% inoculum size. Increase in substrate concentration had a negative effect on growth. Protease activity was maximum at 40 °C (Fig. 3b) and decreased after that. Hence, a range between 40 and 50 °C was selected for RSM design. The levels of pH did not affect the protease production much. Although pH had a slight negative effect on the protease production, in all the runs the final pH obtained was 9 irrespective of the initial pH. Hence, pH 9 was maintained for all the runs in RSM design. Protease activity increased with increasing time and was maximum at 72 h. The protease activity increased linearly with increasing inoculum size and substrate concentration. Among the five factors, three of them, including time, substrate concentration and inoculum size showed a positive effect, but the temperature and pH showed a negative effect.

**Table 3:** The range of variables included in the Plackett-Burman design.

Factors	Units	Minimum level	Maximum level
Temperature	°C	40	80
Time	h	24	72
pH		7	12
Inoculum size	% (v/v)	1	5
Substrate concentration	% (w/v)	0.5	2

**Table 4:** The Plackett-Burman experimental design with the observed growth and protease activity as response variables.

Run	Temperature (°C)	pH	Time (h)	Inoculum size (%)	Substrate concentration. (%)	Growth (660 nm)	Activity (U/ml)
1	40	12	24	1	0.5	0.019	1.64
2	80	12	24	5	2	0.007	4.20
3	80	12	24	5	0.5	0.204	2.16
4	80	7	72	1	0.5	0.007	2.20
5	40	7	24	5	2	0.318	17.04
6	80	7	72	5	0.5	0.007	2.20
7	40	12	72	1	2	0.242	42.24
8	80	12	72	1	2	0.007	2.20
9	40	12	72	5	0.5	0.650	37.52
10	80	7	24	1	2	0.062	2.20
11	40	7	24	1	0.5	1.139	29.24
12	40	7	72	5	2	0.700	50.16

### Optimization by RSM

The range of variables used in response surface design is presented in Table 5. The RSM was performed in 31 runs with four factors (temperature, time, inoculum size and substrate concentration) using the central composite design. Though temperature had a negative effect, it was included for RSM to find out the exact temperature at which the bacterium could produce maximum protease activity. All the runs were conducted at pH 9. The application of the response surface methodology for the obtained data yielded the following quadratic equation in coded units.

**Table 5:** The range of variables included in response surface design.

Factors	Units	Minimum level	Maximum level
Temperature	°C	40	50
Time	h	65	75
Inoculum size	% (v/v)	4	6
Substrate concentration	% (w/v)	1.5	3

Protease activity (U/ml) = −2583 + 43.6 a + 39.5 b + 66.9 c + 59.1 d − 0.4883 a^2^−0.2789 b^2^−5.74 c^2^ − 15.01 d^2^ + 0.006 a*b − 0.107 a*c + 0.625 a*d − 0.018 b*c + 0.021 b*d − 1.90 c*d,

Where, a-temperature, b-time, c-inoculum size and d-substrate concentration.

**Figure 4:**
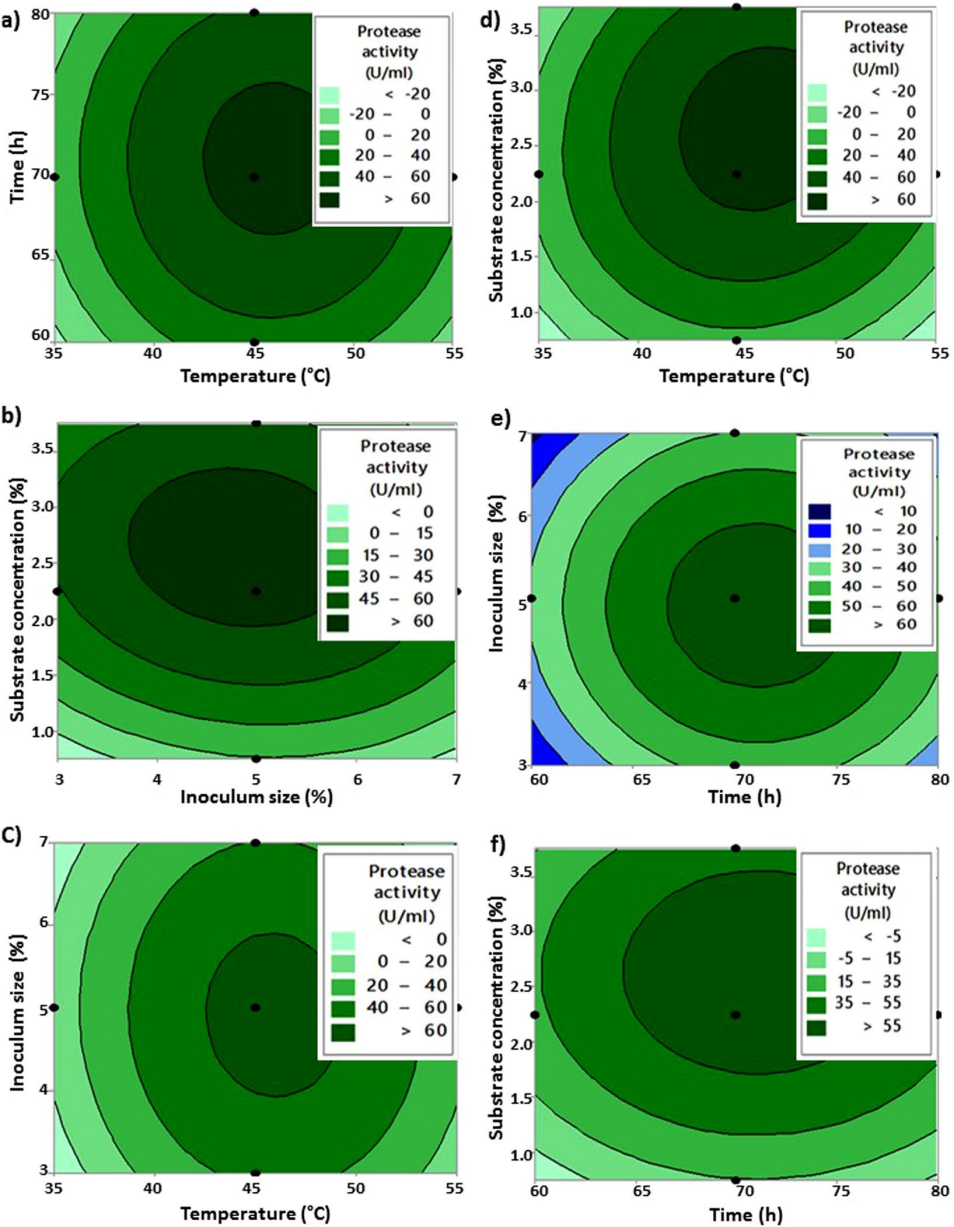
Contour plot of protease activity with interaction between (**a**) temperature and time, (**b**) inoculum size and substrate concentration, (**c**) temperature and inoculum size, (**d**) temperature and substrate concentration, (**e**) time and inoculum size and (**f**) time and substrate concentration. Hold values: Inoculum size 5%, substract concentration 2.25%, time 70 h, and temperature 45 °C.

The runs and the corresponding observed and predicted response variables are given in Table 6. The adequacy of the quadratic model fitting of data was tested by ANOVA and the low P value (0.001) indicates a high significance in the regression model. The R^2^ value of 82.92% confirmed a satisfactory adjustment of the quadratic model to the experimental data and suggested that there is no significant difference between the predicted and experimental values, thus, making the model acceptable to optimize the conditions for protease^17^. Contour plots were plotted to show the effect of two factors on the protease activity, keeping other factors at constant central values (Fig. 4a–f). The best conditions for protease production observed from contour plots were temperature 45 °C, incubation time of 70 h, inoculum size 5% and substrate concentration 2.3%. Using these experimental results, the optimum conditions for protease production were predicted using Minitab as temperature 46.31 °C, incubation time of 71.11 h, inoculum size 4.86% and substrate concentration of 2.66%. The predicted maximum protease activity was 68.56 U/ml. The validation experiment of the model optimization was carried out. There was no significant difference between the observed (73.52 U/ml) and predicted protease activity using a t-test at the 5% level (t-test; P > 0.05). Thus, the model was accepted and the same conditions were used for the final protease production. The experiment under optimized conditions showed a 1.5-fold increase in protease production. A study reported by Beg *et al*.^18^ showed that alkaline protease production by *Bacillus mojavensis* was increased up to 4.2 fold in a 14 L bioreactor using RSM. In another study, there was an overall increase of 2.3 fold in protease production by *Bacillus* sp. RKY3 after optimization^19^. Cost-effective media formulation and statistical optimization are very useful for maximum enzyme production in laboratory and industrial-scale enzyme production. The optimum temperature 46.31 °C for the protease indicates that the produced alkaline protease is thermotolerant in nature.

**Table 6:** Response surface methodology runs with the response variable protease activity (observed and predicted) and the predicted protease activity using artificial neural network.

Run	Temperature (°C)	Time (h)	Inoculum (%)	Substrate concentration (%)	Observed activity (U/ml)	Predicted activity using RSM (U/ml)	Predicted activity using ANN (U/ml)
1	50	65	4	1.5	25.68	21.96	25.68
2	45	70	5	2.25	63.4	65.14	64.84
3	50	75	6	3	59.64	47.79	59.44
4	45	70	5	2.25	63.32	65.14	64.84
5	45	70	5	2.25	64.84	65.14	64.84
6	40	75	4	3	38.24	38.49	27.54
7	45	70	3	2.25	40.8	43.91	40.45
8	40	75	4	1.5	19.6	22.80	19.60
9	45	70	5	3.75	33.72	48.73	33.89
10	50	65	6	1.5	27.44	22.21	27.41
11	40	65	4	3	30.16	32.16	30.16
12	45	80	5	2.25	33.08	43.53	33.07
13	50	75	4	3	66.68	53.62	66.47
14	40	65	6	3	29.88	28.85	22.25
15	40	65	6	1.5	10.64	19.18	10.55
16	50	75	4	1.5	32.52	28.57	32.23
17	40	65	4	1.5	9.92	16.79	9.80
18	45	70	5	2.25	64.88	65.14	64.84
19	40	75	6	3	35.6	34.80	35.60
20	55	70	5	2.25	0.48	25.39	—
21	50	75	6	1.5	34.96	28.45	35.06
22	50	65	4	3	54	46.71	53.87
23	45	70	7	2.25	34.08	40.47	43.68
24	50	65	6	3	48.96	41.24	48.84
25	40	75	6	1.5	22.52	24.83	16.00
26	45	70	5	2.25	66.56	65.14	64.84
27	45	70	5	2.25	66.68	65.14	64.84
28	45	70	5	0.75	19.52	14.01	19.34
29	35	70	5	2.25	22.64	7.22	14.52
30	45	70	5	2.25	66.28	65.14	64.84
31	45	60	5	2.25	31.92	30.97	31.90

Sum = 11.0418, R^2^ = 82.92%, R^2^ (adjusted) = 67.97%. Each value is mean of triplicate values. Using ANN R^2^ = 96.68%.

### ANN

**Figure 5:**
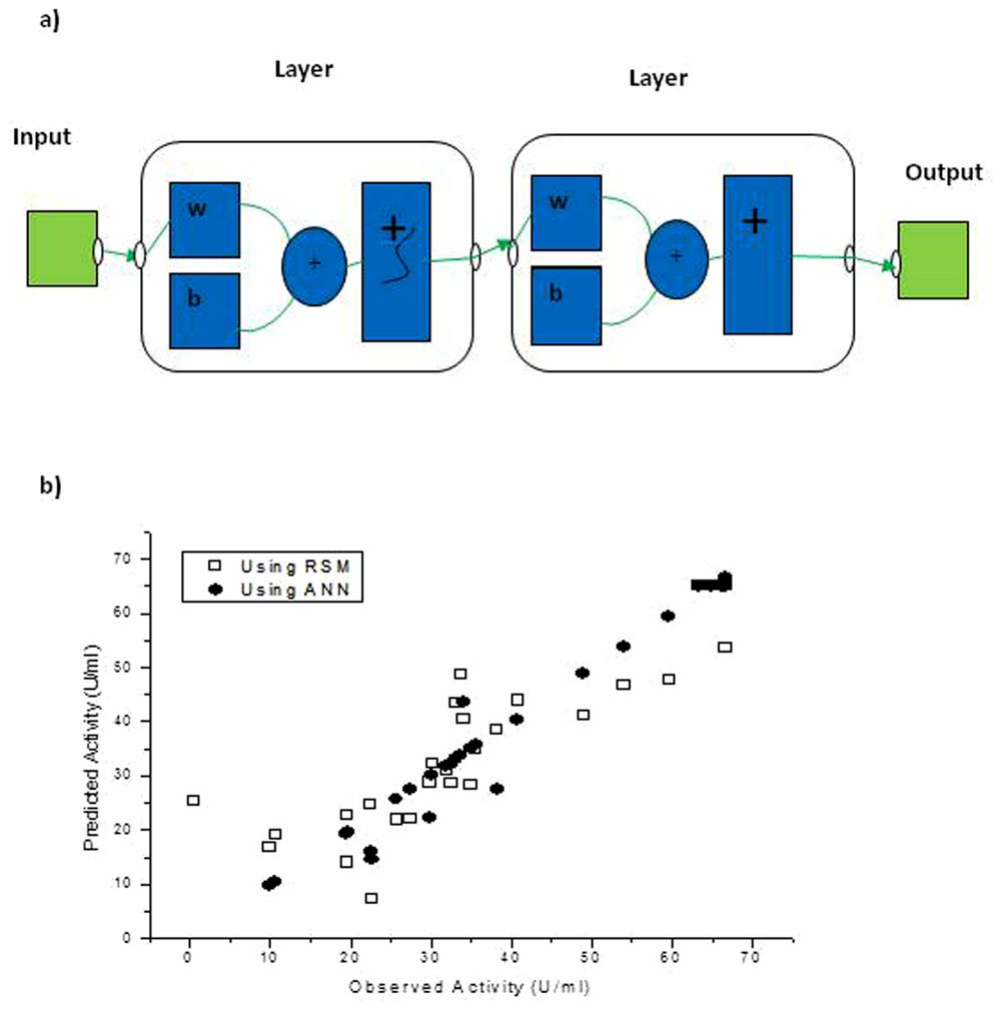
(**a**) Input-output structure of Artificial Neural Network and (b) Observed and predicted activity using RSM and ANN

The ANN is a black box concept based model which involves training, validation and testing structure and functionalities of biological neural networks. Artificial neuron (in terms of black box based simple artificial function) is a fundamental concept of ANN^20^. Such a model has three simple set of rules: multiplication, summation and activation. Inputs are weighted at the entrance of artificial neurons, sum function that sums all weighted inputs and bias performed in the middle section of artificial neuron. At the exit of artificial neuron, the sum of formerly weighted inputs and bias is passing through an activation function called the transfer function (Fig. 5a). The overall coefficient of determination (R^2^) value of 82.2% using RSM method encouraged us to make use of ANN to obtain a better model with higher accuracy in R^2^. ANN has been proven to be a successful tool in black-box based studies by a large number of researchers^20^ and their improved results motivated us to gain further understanding of protease activity with respect to its associated independent decision variables. The detailed working mechanism of ANN along with information on its control parameters, number of hidden layers, transfer function, algorithm used for training, etc. is summarized below along with improved results.

In the existing problem, initially the data consisting of 31 data points having four variables data (namely, temperature, time, inoculum and substrate concentration) and the corresponding overall activity were considered. The data were filtered and one data point corresponding to the lowest extreme value of overall activity of 0.48 was discarded from ANN analysis. Thus, in total 30 numbers of data points were considered, Among the 30 points, 20 randomly chosen points were used for training purpose, 5 points were used for validation and the remaining 5 individual solutions were used for testing the neural network. ‘Nntraintool’ toolbox from Matlab 2011 library was used. The number of hidden layers was varied and several trial runs were carried out. Finally, it was observed that with 12 numbers of hidden layers, the best possible predictions were obtained. Levenberg-Marquardt algorithm was used for training purpose. The mean squared error (MSE) criteria were used to test performance of predicted data. Random algorithm was used for data division. The mean squared error (MSE) for testing was observed to 0.29 (for 20 sets of data points), whereas for validation and testing, the corresponding MSE was 32.62 and 43.46 respectively (for each 5 sets of data points). The Table 6 and Fig. 5b shows the predicted values of protease activity obtained using ANN. Figure, 5b also shows the comparative plot between the observed and predicted activity as obtained using RSM and ANN methods. The figure clearly shows that using the black box based ANN method using Levenberg-Marquardt algorithm for training purpose resulted in solutions such that the majority of the solutions complement each other in terms of observed and predicted activity when ANN was used. The overall coefficient of determination (R^2^) value using ANN is 96.67%, which is higher than the 82.2% obtained by RSM method. Thus, ANN is observed to be an improved tool to predict the experimental results.

### Partial purification and molecular weight determination

**Figure 6:**
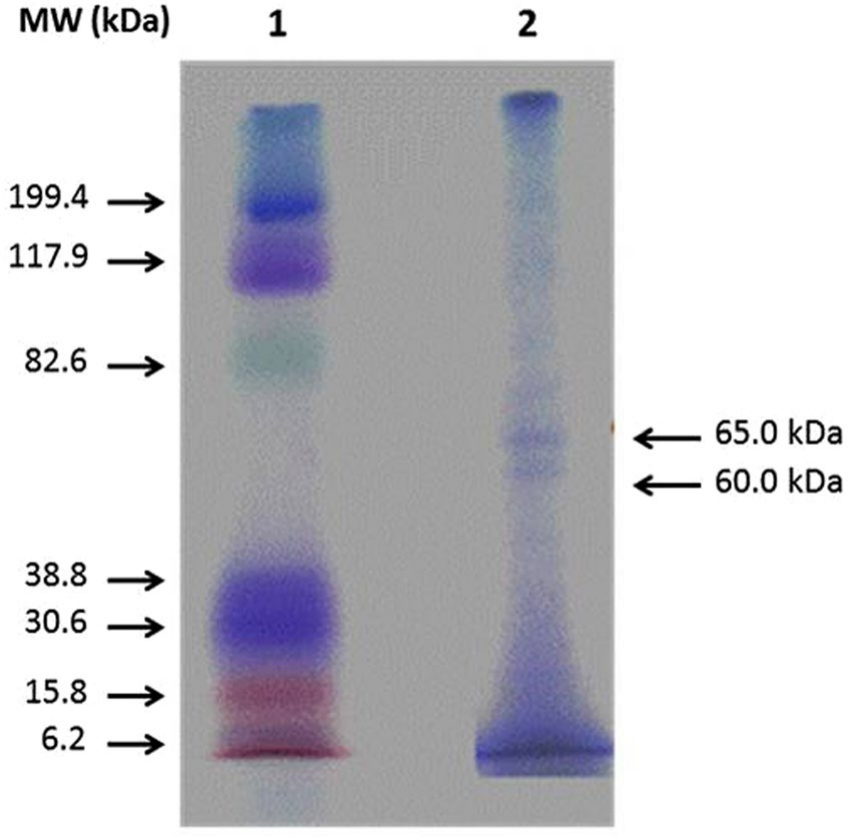
SDS-PAGE analysis of the purified protease from *Bacillus licheniformis* NK. Lane 1: protein markers, Lane 2 (cropped): purified protease by Sephadex G-100 column.

The stages of purification and the corresponding protease activities are given in Table 7. After purification, the activity increased by 6.5-fold. The purified enzyme produced two bands each corresponding to molecular weight 60 and 65 KDa (Fig. 6; Supplementary Fig. 6). Two band formation could be due to the presence of two types of proteases^21^ or single enzyme with two polypeptide chains^22^.

**Table 7:** Stages of purification and corresponding activity and yield of protease enzyme.

Purification stage	Volume (ml)	Total protein (mg)	Activity (U/ml)	Total activity (U)	Specific Activity (U/mg)	Fold	Yield (%)
Crude supernatant	224	902.804	73.51	16467.30	18.24	1.00	100.00
Ammonium sulphate fraction	3	14.786	116.33	349.00	23.60	1.29	2.12
Dialyzed sample	2	10.929	238.40	476.80	43.63	2.39	2.90
Sephadex column	6	2.379	46.72	280.32	117.83	6.46	1.70

### Characterization of the protease

#### Effect of pH

**Figure 7:**
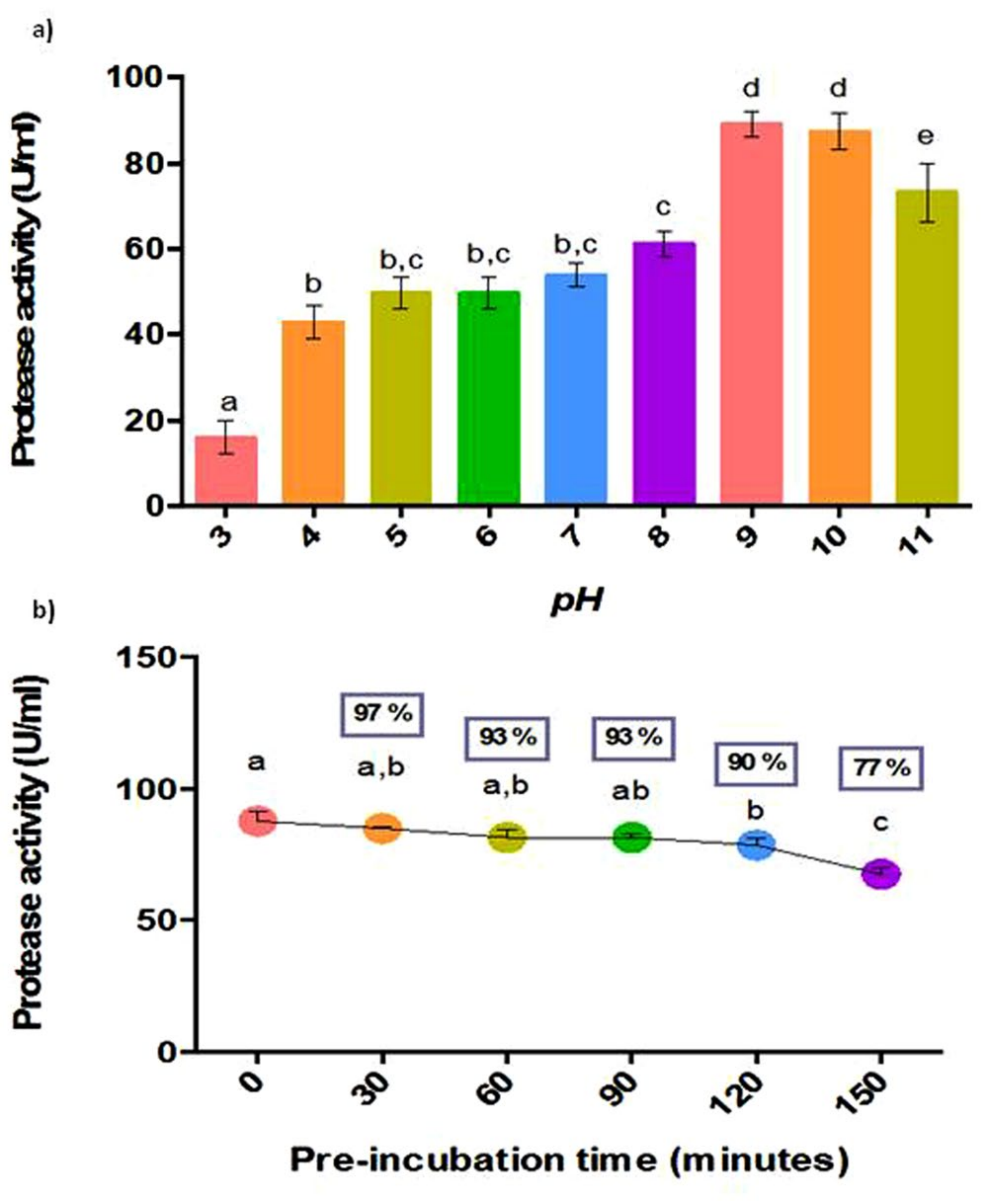
**(a**) Effect of pH on protease activity and (b) Stability of protease at pH 9. Bars with same alphabets are not significantly different from each other (Turkey’s test; P < 0.05). Residual activity (%) compared to control (0 min) is given within the box above each point.

The optimum pH range for alkaline proteases generally ranges from 9–11. The purified protease from *B*. *licheniformis* NK exhibited a reasonable activity in a pH range of 5–11 with a significantly higher activity at pH 9 and 10 (Fig. 7a). It retained 98% activity at pH 10 and 82% at pH 11. Though it is alkaline, the protease retained 57% activity at pH 5. The stability of the enzyme was measured at pH 9 by incubating the enzyme for 150 min and measuring the activity at every 30 min interval. The enzyme was stable up to 90 min at pH 9 with a residual activity of 93% (Fig. 7b). The protease retained 90% activity, even at 120 h at pH 9. Thus, the activity of protease produced from *B*. *licheniformis* NK is comparable to the commercialized detergent enzymes such as Maxatase (Gist-brocades, The Netherlands), produced by *B*. *subtilis* having an optimum range between pH 9 and 10^23^. The broad pH range of activity, its optimum activity at higher pH values and the stability for a longer duration at alkaline pH suits this enzyme highly attractive for detergent industry, since laundry detergents generally operate at a pH of 7–11 and other industrial processes involving alkaline processes. Apart from detergent industries, alkaline proteases are also utilized in the formulation of household dishwashing and cleaning detergents, preparation of protein hydrolysates, dehairing and bating of skin and hides during leather processing^24^.

#### Effect of temperature

**Figure 8:**
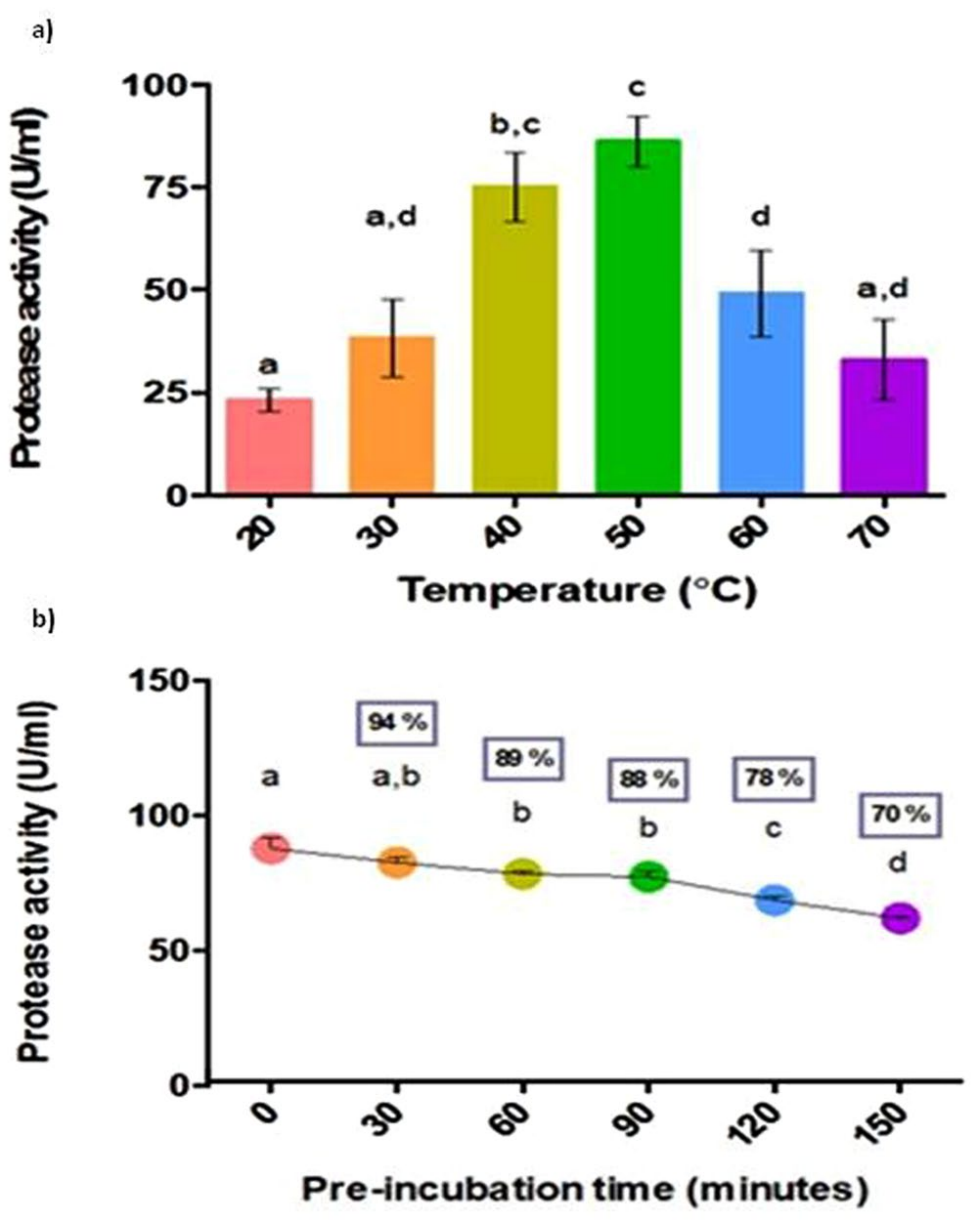
(**a**) Effect of temperature on protease activity and (**b**) Stability of protease at temperature 50 °C. Bars with same alphabets are not significantly different from each other (Turkey’s test; P < 0.05). Residual activity (%) compared to control (0 min) is given within the box above each point.

Alkaline protease from *B*. *licheniformis* NK showed a significantly higher activity at 50 °C and a reasonable activity was observed between 40 to 60 °C (Fig. 8a). Yang *et al*.^15^ reported that the protease from *B*. *subtilis* also showed maximum activity at 50 °C. The stability of the protease was studied at 50 °C. The protease maintained a residual activity of 94% at 30 min at 50 °C (Fig. 8b) and retained 70% residual activity, even at 150 min. The produced protease had a residual activity of 89% after one hour at 50 °C, which was much higher than 30% of residual activity reported by Banik *et al*.^25^. Proteases used in detergent industries should have high activity at alkaline pH ranges and should be thermostable in order to be effective during washing. The protease produced in this study is alkaline in nature and thermotolerant and therefore has the potential for detergent industry applications. In addition, thermostable enzymes can minimize the problem of contamination by running the processes not suitable for mesophilic microbes. This property would be very helpful when fish gut waste is used as a substrate for protease production. Further, the high temperature increases the substrate solubility and lower the viscosity of liquids which helps in mixing of medium components.

#### Effect of metal ions

**Figure 9:**
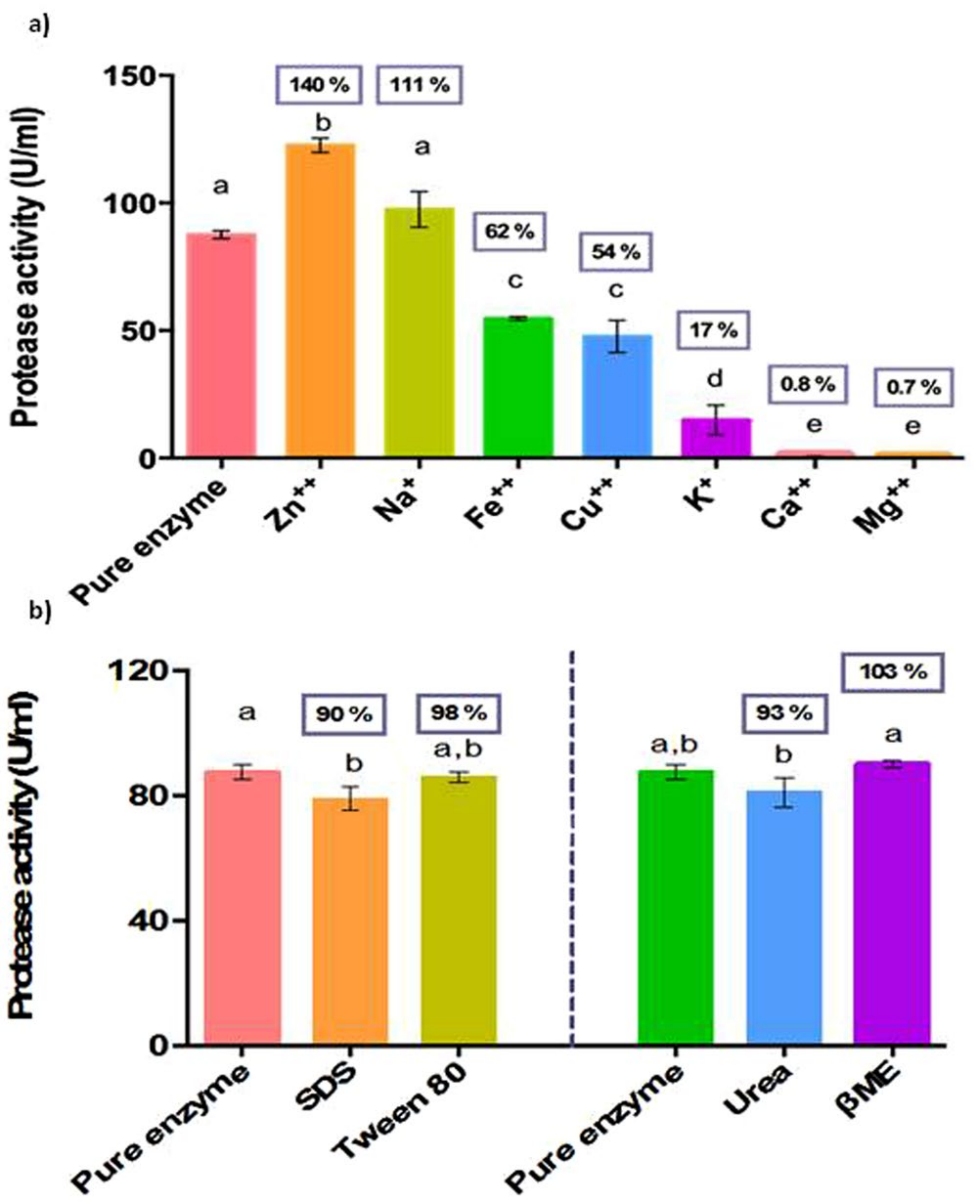
Influence of (**a**) metal ions (10 mM), (**b**) surfactants (1%) and inhibitors (2.5 mM) on protease activity at pH 9 and temperature 50 °C. Bars with same alphabets are not significantly different from each other (Turkey’s test; P < 0.05). Residual activity (%) compared to the activity of pure enzyme is given within the box above each bar.

The effect of different metal ions on protease activity was examined (Fig. 9a). The inducers Zn^++^ and Na^+^ increased the activity by 40% and 11% respectively. Binding sites of Zn^++^ and Na^+^ may stabilize and activate the enzyme. However, Fe^++^, Cu^++^, K^+^, Ca^++^, and Mg^++^ inhibited the activity. Abou-Elela *et al*.^26^ reported that protease produced from *Bacillus cereus* MCM B-326 was completely inhibited by Mg^2+^ and Zn^2+^ and partially inhibited by Na^+^, whereas Ca^2+^ was not required for activity but was essential for growth^22^. In contrast, it was reported that Mn^2+^, Ca^2+^ and Mg^2+^ ions have increased the relative protease activity of *Bacillus megaterium* isolated from Thai fish sauce^27^. Following the addition of MnCl_2_ and CaCl_2_, the activity of alkaline protease from *Trametes cingulata* strain CTM10101 was enhanced by 170 and 219%, respectively^28^. This shows that there is no standard cofactor for enhancement of protease activity. The enzyme produced by different strains of bacteria of diverse locations may require different metal ions to trigger their action.

#### Effect of surfactants and inhibitors

Inhibition studies give an idea about the nature of the enzyme, its active center and its cofactor requirements. The alkaline protease was stable against SDS and Tween 80 by showing residual activity of 90 and 98% respectively (Fig. 9b). The stability towards SDS is important in detergent industry as SDS is a very common additive in commercial detergents and *Bacillus* spp. are known to be the most common producers of such stable enzymes. The protease enzyme of this study showed a residual activity of 93 and 103% against urea and βME. Urea is a common surfactant used in detergents. βME is an aspartic protease inhibitor^29^, and it did not have any effect on the enzyme, indicating that this enzyme may not be an aspartic protease.

#### Compatibility with commercial detergents

**Figure 10:**
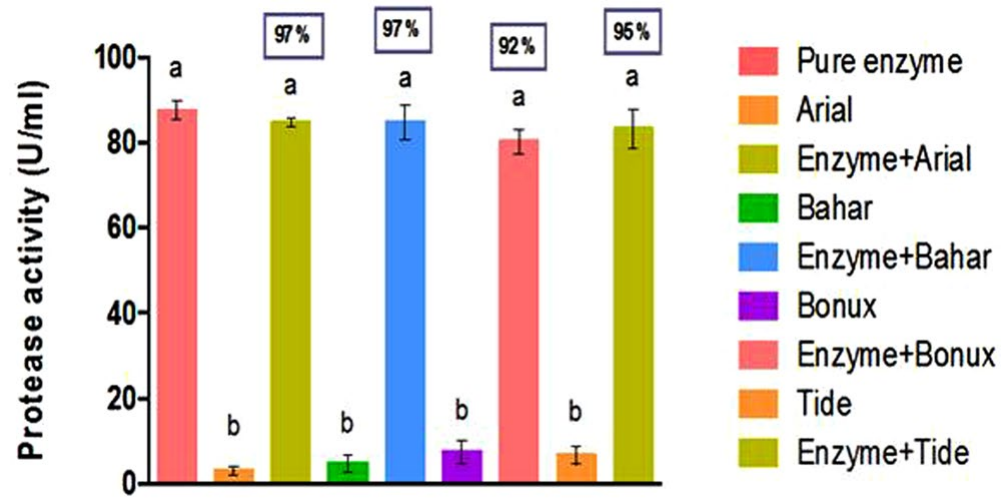
Compatibility of protease with commercial detergents (0.1% w/v). Bars with same alphabets are not significantly different from each other (Turkey’s test; P < 0.05). Residual activity (%) compared to the activity of pure enzyme is given within the box above each bar.

The purified protease is compatible with all the commercial detergents used and the residual activity was above 90% in all the detergents (Fig. 10). Maximum residual activity was observed with Arial and Bahar (97%), followed by Tide (95%) and Bonux (92%). The small bars show the protease activity in the respective commercial detergents available in the market. It has been reported that the alkaline protease produced from *B*. *licheniformis* RP1 retained 95% of its initial activity with Arial followed by Axion (94%) and then Dixan (93.5%) when pre incubated with detergents at 40 °C for 60 min^30^. A serine alkaline protease from *Bacillus* sp. SSR1 exhibited almost 70–80% of activity in most of the detergents at 40 °C^31^. The detergent compatibility of the alkaline protease produced in this study is higher than the previously reported enzymes, which suits the enzyme as a best additive to the detergents.

#### Substrate specificity

**Figure 11:**
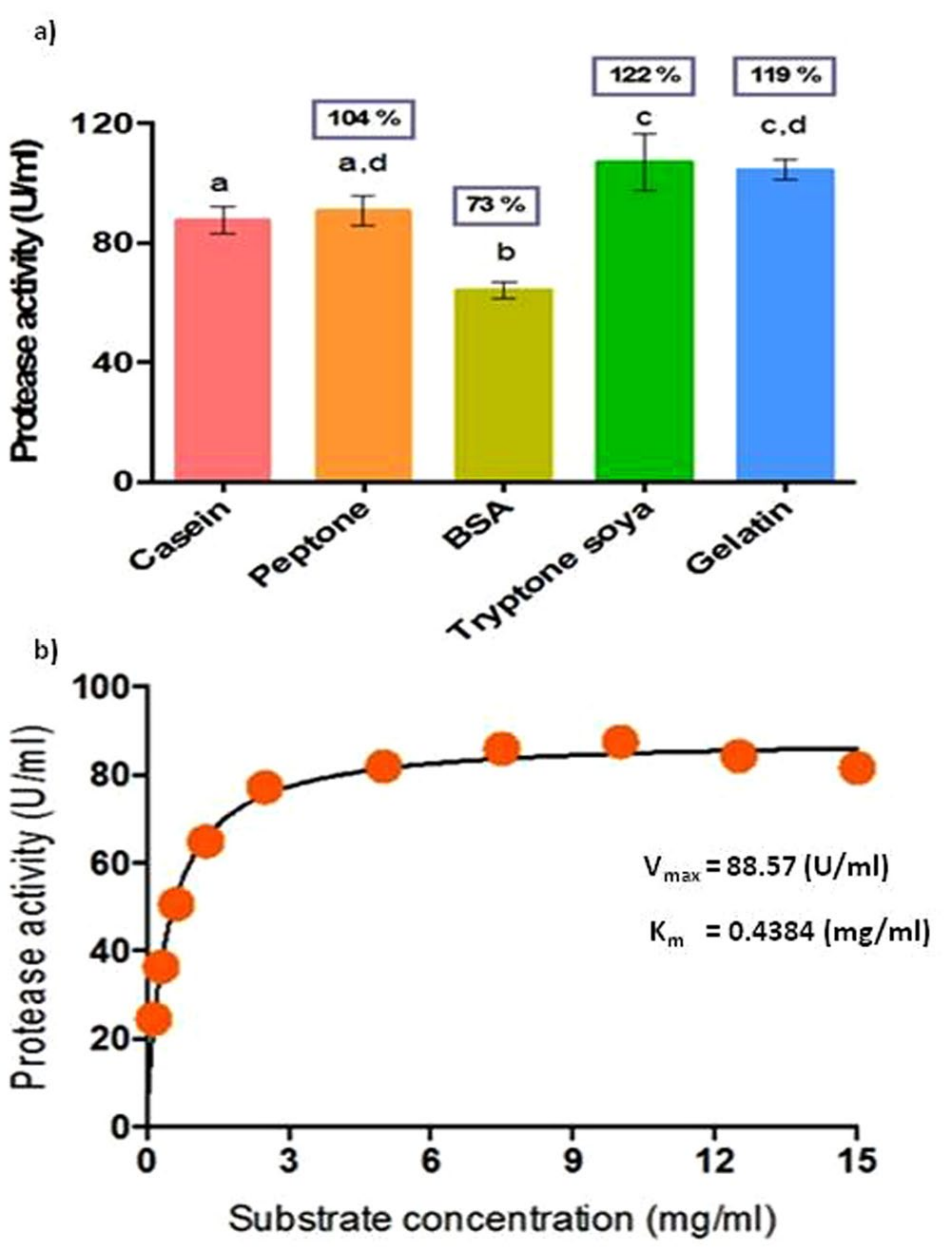
Effect of (**a**) Different substrates (1%) on protease activity. Protease activity in the presence of casein was considered 100%. Bars with same alphabets are not significantly different from each other (Tukey’s test; P < 0.05). Residual activity (%)compared to the activity of pure enzyme is given within the box above each bar.(**b**) Substrate concentration on protease activity using non-linear regression and estimation of enzyme kinetic parameters.

Different substrates such as peptone, BSA, tryptone soya and gelatin (1%) were used to compare their influence on protease activity as opposed to that of the pure enzyme with casein. Results showed that tryptone soya, gelatin and peptone are suitable substrates having activity 122, 119 and 104% respectively, whereas with BSA the activity decreased to 73% (Fig. 11a). The results suggested that this protease is able to digest various protein substrates and could convert them into small peptides and amino acids, demonstrating that this alkaline protease has a broad range of substrate specificity. The protein hydrolysates obtained from casein, soy protein and whey protein are useful in the formulation of hypoallergenic infant food. Further, they can also be useful in fortification of fruit juices and in preparation of high protein therapeutic diets^32^. In addition to its stability at different pH, temperature, surfactants, inhibitors and detergents, this wide range of substrate utilization makes the enzyme as a suitable candidate for various industrial applications including food industries.

#### Enzyme kinetics

The kinetic parameters, V_max_, the maximum enzyme velocity and K_m_, the Michaelis- Menten constant of the protease enzyme were estimated by measuring the enzyme activity at different concentrations of the substrate casein (0.156–15 mg/ml). The equation describing the relationship is Y = V_max_*X/(K_m_ + X). Figure 11b shows the results of the non-linear regression and the values of V_max_ (88.57 U/ml) and K_m_ (0.4384 mg/ml). The tested null hypothesis is the Michaelis-Menten as opposed the alternate hypothesis of allosteric sigmoidal. The calculated F = 0.6232< tabulated F_0.05, 1,27_ = 4.21 at P = 0.8048 and goodness of fit as R^2^ = 0.9872 accepts the null hypothesis and the best model fitting the present data is Michaelis-Menten. The increase in substrate concentration here increased the protease activity up to 10 mg/ml, after which it is saturated. The smaller K_m_ value indicates the high affinity and efficient catalytic role of the enzyme towards the substrate^33^.

## Conclusion

In this study, gut waste of *S*. *longiceps* has been used as a low cost substrate for protease production using *B*. *licheniformis* NK isolated from Nakhl hot spring, Oman. The best conditions for the protease production were optimized as 46.31 °C, incubation time 71.11 h, inoculum 4.86% and substrate concentration 2.66%. The predicted maximum protease activity was 68.56 U/ml. The validation experiment of the model optimization showed a protease activity of 73.52 U/ml. ANN is a better tool to predict the experimental results. The protease enzyme retains maximum activity and stability at pH 9 and 50 °C. The enzyme was stable against surfactants, inhibitors and compatible with the commercial detergents. This study concludes that fish gut waste is a worthy low cost substrate for protease production and the produced detergent compatible, thermotolerant, alkaline protease has the potential for industrial applications.

## Materials and Methods

### Sampling

Soil and water samples were collected from four different sites from Nakhl hot spring (23°22′32“N; 57°49′39″E) in Oman. Air and water temperatures, pH, conductivity and dissolved oxygen levels were recorded at each site during the time of sampling.

### Preparation and analysis of fish gut waste substrate

Guts from *S*. *longiceps* were excised and washed with water to remove the blood and boiled for 20 min. After draining the excess water, the boiled waste was minced and dried at 50 °C for 72 h in an oven. The dried substance was finely ground to a powder and stored at room temperature. The gut waste was analyzed for its total protein concentration^34^, total lipids^35^ and total carbohydrates^36^ on dry weight basis. Chemical elements in the gut waste were analyzed by inductively coupled plasma mass spectrometry (ICP-MS, BRUKER Aurora M90, USA) and compound anions were estimated by Ion Chromatography (IC, 850 Professional, Metrohm, Switzerland).

### Screening and identification of protease producing bacteria

Soil and water samples collected from Nakhl hot spring were inoculated into casein agar plates (g/l: casein 2, glucose 2, K_2_PO_4_ 0.2, MgSO_4_ 0.2, FeSO_4_.7H_2_O 0.01, and agar 20). Protease producing bacteria were selected based on the size of the clearance zone formed around the colonies. Secondary screening was carried out in 1% fish gut waste broth medium (without any additives), with 4% (v/v) inoculum and incubated at 50 °C in order to identify the best protease producing bacteria. The bacterial strains that showed positive results in secondary screening were identified by matrix-assisted laser desorption/ionization (MALDI) biotyper (BRUKER, Microflex). The best protease producing bacteria after secondary screening was identified by 16 S rRNA sequencing.

### Protease assay

Protease activity was measured by the modified method of Puri *et al*.^17^ using casein as a substrate and tyrosine as a standard. To 0.25 ml of sample, 0.25 ml of 1% casein in 50 mM glycine NaOH buffer (pH 9) was added and incubated for 10 min at 50 °C. To stop the reaction 0.5 ml of 0.4 M TCA was added and the mixture was centrifuged at 10000 rpm for 10 min. From the supernatant 0.5 ml was taken and to this 2.5 ml of 0.4 M sodium carbonate and 0.25 ml of 2 N Folin’s reagent was added and incubated for 30 min at room temperature. Absorbance was read at 660 nm using a spectrophotometer (Thermo spectronic, USA). One unit of protease activity is defined as the amount of enzyme required to liberate 1 µmole of tyrosine under defined standard assay conditions.

### Optimization and production

#### Screening of significant factors by PB design

The PB design is an effective way to find factors with positive effects^37^ on protease production among the number of variables used. The PB design is a two level fractional factorial screening design for studying N-1 variables using N runs, where N is a multiple of four. Each factor was studied at two levels, −1 for a low level and + 1 for a high level. The variables selected for this study were temperature, time, pH, percent of inoculum (v/v) and percent substrate concentration (w/v) (Table 3). Totally, 12 runs were generated in PB design.

### Optimization by RSM

Based on the results obtained from PB runs, a response surface design was generated to determine the optimum levels of the selected variables in protease production^37^. In RSM, 31 experiments were conducted in triplicate using central composite design. The factors considered for RSM designs are given in Table 5. The protease activity, the response variable in the model generated, was statistically analyzed and conditions for maximum protease activity were predicted. A validation experiment was conducted in triplicate to confirm the prediction. Both PB and RSM were designed using the statistical software Minitab, Version 17.

### ANN

Thirty-one data sets having four variables, temperature, time, inoculum size and substrate concentration and the corresponding overall activity were considered for ANN study. The data were filtered and the lowest activity of 0.48 was not included in ANN analysis. Out of 30 available data sets, 20 were used for training purpose, 5 used for validation and the remaining 5 to test the neural network. ‘Nntraintool’ toolbox from Matlab 2011 library was used.

### Enzyme production and partial purification

After the fermentation, the cells were removed from the crude supernatant by centrifugation at 10000 rpm for 10 min. The protein in the supernatant was precipitated by 60% ammonium sulphate and dialyzed against Tris HCl buffer. The dialyzed sample was loaded onto a Sephadex G-100 column pre-equilibrated with 25 mM Tris HCl eluting buffer (pH 7) and was eluted at a flow rate of 0.2 ml/min through the column. The eluted fractions with the highest activity were pooled and used for characterization.

### Characterization

#### Molecular weight determination

The molecular weight of the purified protease was determined using sodium dodecyl sulphate polyacrylamide gel electrophoresis (SDS-PAGE) using a 12% polyacrylamide gel. Kaleidoscopic prestained low molecular weight protein standard (6.2 KDa – 199.4 KDa) from BIORAD was used as the ladder.

#### Effect of pH on the activity and stability

To evaluate the effect of pH on protease, the activity of the purified protease was measured at different range of pH (3–11). The buffers used for different pH ranges were glycine HCl (pH 3–4), phosphate buffer (pH 5–6), Tris-HCl buffer (pH 7–8) and Glycine NaOH (pH 9–11). The pH at which the protease showed high activity was considered as optimum and the pH stability of the enzyme was determined by measuring the protease activity at every 30 min interval up to 2.5 h by incubating the enzyme at the optimum pH. Residual activity was measured and the activity measured without pre-incubation was considered as the control with 100% activity.

#### Effect of temperature on the activity and stability

The effect of temperature on the protease activity has been studied by incubating the protease at various temperatures (20–70 °C) for 30 min at optimum pH. Thermal stability was studied by incubating the protease for 150 min at optimum temperature. Aliquots were withdrawn at 30 min intervals to test the residual activity at optimum conditions. The activity measured without pre-incubation was considered as the control with 100% activity and the above optimum pH and temperature were used for the remaining characterization assays.

#### Effect of metal ions, surfactants, inhibitors, detergents and substrates

The effect of some metal ions (10 mM) such as Zn^++^, Na^+^, Fe^++^, Cu^++^, K^+^, Ca^++^ and Mg^+^, surfactants (0.1%) such as sodium dodecyl sulphate (SDS) and Tween 80 and 2.5 mM of inhibitors such as β-mercaptoethanol (βME) and urea were used to study their effect on protease activity. Effect of commercial detergents (0.1% w/v) such as Arial, Bahar, Bonux and Tide were used to study their effect on protease enzyme. The influence of various substrates (1%) like peptone, bovine serum albumin (BSA), tryptone soya and gelatin were used to study their effect on protease activity. For all experiments, the enzyme activity was studied by pre-incubating the protease enzyme with the respective component for 30 min at room temperature. Residual activity after 30 min was measured and activity without pre-incubation was considered as 100% activity.

### Determination of V_max_ and K_m_ values

The enzyme kinetic parameters, V_max_ and K_m_ values of the protease enzyme were estimated by measuring the protease activity using different concentrations of substrate (0.156–15 mg/ml). V_max_ and K_m_ values were determined using the Michaelis-Menten equation through nonlinear regression analysis using the statistical software Graphpad PRISM version 6.

### Statistical analysis

All the experiments were conducted in triplicate. Significant changes in the mean values were analyzed by one-way ANOVA followed by Tukey’s Post Hoc analysis using IBM SPSS statistics software version 21. Statistical difference at p < 0.05 were considered as significant.

### Data availability

All data generated or analyzed during the study are included in this article.

## Electronic supplementary material

Supplementary Information
