# Supplementary material for: Production of thermotolerant, detergent stable alkaline protease using the gut waste of Sardinella longiceps as a substrate: Optimization and characterization
Source: Sci Rep. 2018 Aug 20;8:12442. doi: 10.1038/s41598-018-30155-9 (PMC6102305; doi:10.1038/s41598-018-30155-9)
Supplement: Supplementary file 1 — Supplementary Information [file 41598_2018_30155_MOESM1_ESM.pdf]

Production of thermotolerant, detergent stable alkaline protease using the gut waste of *Sardinella longiceps* as a substrate: Optimization and characterization

Aishwarya Ramkumar, Nallusamy Sivakumar *, Ashish M. Gujarathi, Reginald Victor

[1] Department of Biology, College of Science, Sultan Qaboos University, PO Box 36, PC 123, Muscat, Oman

[2] Department of Petroleum and Chemical Engineering, College of Engineering, Sultan Qaboos University, Muscat, Sultanate of Oman

*Corresponding author's email: apnsiva@squ.edu.om

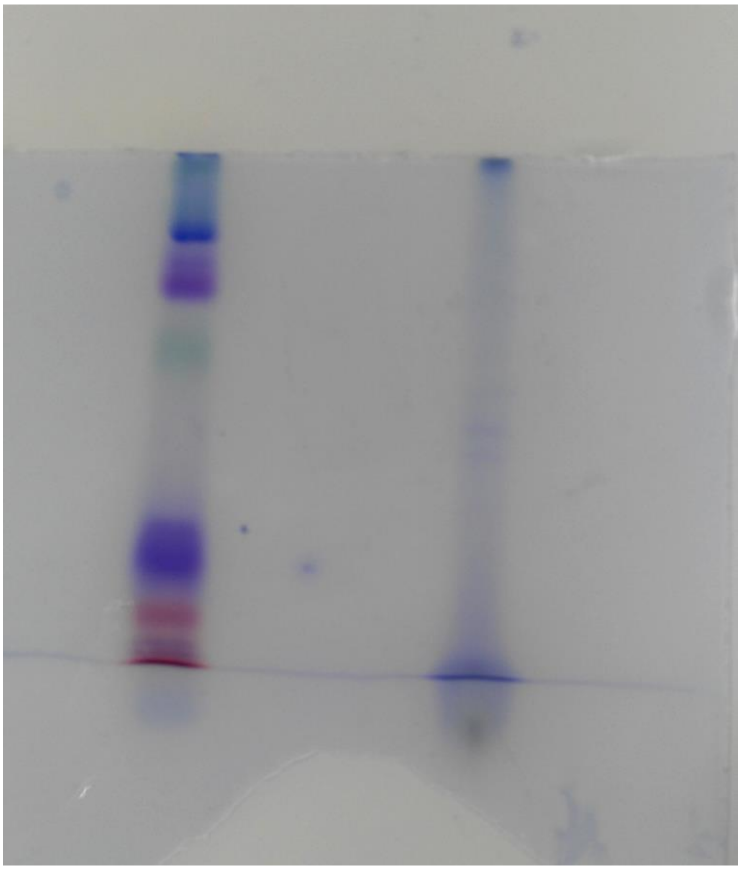

Figure 6. The original photograph of SDS-PAGE. Only two lanes were loaded. Lane 1: protein markers, Lane 2: Protease from *Bacillus licheniformis* NK.
